# Targeted expression of the immunomodulatory peptide Scy by engineered *Escherichia coli* Nissle 1917 for alleviating enterotoxigenic *E. coli* infection

**DOI:** 10.3389/fimmu.2026.1698243

**Published:** 2026-02-12

**Authors:** Jie Fu, Lutong Zhou, Xiao Jiang, Hong Zhang, Yanli Chen, Lei Qiao, Deguang Song, Zeqing Lu, Tenghao Wang, Mingliang Jin, Caiqiao Zhang, Yizhen Wang, Yuanzhi Cheng

**Affiliations:** 1 Key Laboratory of Molecular Animal Nutrition, Ministry of Education, Zhejiang University, Hangzhou, Zhejiang, China; 2 Department of Veterinary Medicine, College of Animal Sciences, Zhejiang University, Hangzhou, Zhejiang, China; 3 Key Laboratory of Animal Nutrition and Feed Science (Eastern of China), Ministry of Agriculture, Zhejiang University, Hangzhou, Zhejiang, China; 4 Zhejiang Key Laboratory of Nutrition and Breeding for High-Quality Animal Products, Zhejiang University, Hangzhou, Zhejiang, China; 5 School of Medicine, Department of Immunobiology, Yale University, New Haven, CT, United States; 6 Zhejiang Qinglian Food Co., Ltd., Jiaxing, Zhejiang, China

**Keywords:** engineered probiotic, *Escherichia coli* Nissle 1917, *ETEC* K88, intestinal immunity, microbiota-based intervention, peptide delivery, SCY

## Abstract

Oral delivery of functional small peptides holds great promise for the prevention and treatment of intestinal diseases. However, their clinical application is limited by rapid degradation in the gastrointestinal tract, low bioavailability, and poor targeting capacity. To address these challenges, we engineered the probiotic *Escherichia coli* Nissle 1917 to express the immunomodulatory peptide Scy (EcN-Scy) under the control of the anaerobically responsive pNirB promoter, thereby enabling site-specific expression in the hypoxic intestinal environment. *In vitro* experiments demonstrated that EcN-Scy exhibited stable peptide expression and strong tolerance to heat, acid, bile salts, and simulated gastrointestinal fluids. *In vivo*, EcN-Scy administration caused no organ toxicity or metabolic abnormalities in mice, but significantly enhanced colonic antioxidant capacity, corrected the imbalance of Th1/Th2 cytokines, and maintained local immune homeostasis. 16S rDNA sequencing revealed that EcN-Scy reshaped the gut microbiota structure by increasing the abundance of beneficial genera such as Parabacteroides, while suppressing the proliferation of opportunistic pathogens including *Alistipes* and *Muribaculaceae*. In the enterotoxigenic *Escherichia coli* (ETEC) K88 infection model, EcN-Scy significantly alleviated diarrheal symptoms and colonic mucosal injury, enhanced the expression of barrier-associated proteins, and suppressed pro-inflammatory cytokine release in the gut. Compared with wild-type EcN, EcN-Scy exhibited markedly superior effects in anti-inflammatory, anti-infective, and barrier-protective functions. Collectively, EcN-Scy represents a novel peptide-producing engineered probiotic capable of targeted intestinal delivery and localized immune modulation, offering substantial potential as an oral microecological therapeutic for the management of intestinal inflammation and pathogen-induced infections.

## 1 Introduction

The intestinal tract is a pivotal organ for nutrient absorption and immune regulation in animals. Its structural integrity, immune homeostasis, and microbial composition are fundamental to maintaining host health (1–4). In recent years, the rising incidence of inflammatory bowel diseases, infectious diarrhea, and microbiota-related disorders in both humans and animals has underscored a critical scientific question in life sciences and veterinary medicine (5–11): how can we effectively intervene in the intestinal microecology and immune environment? Functional bioactive peptides, particularly small peptides with immunomodulatory and anti-inflammatory properties, have attracted growing interest as promising alternatives to antibiotics due to their defined mechanisms of action and minimal toxicity.

Scy is a novel immunomodulatory peptide derived from marine actinomycetes (12). Previous studies have demonstrated its potent anti-inflammatory, antimicrobial, and antioxidant activities (12–15). Notably, Scy has shown significant efficacy in suppressing pro-inflammatory cytokine expression, modulating cytokine networks, and promoting epithelial repair. As a non-traditional peptide-based therapeutic candidate, Scy offers a promising biological foundation for developing novel strategies to manage intestinal disorders (16, 17). However, as a linear peptide, Scy is highly susceptible to proteolytic degradation in the gastrointestinal tract, resulting in poor oral bioavailability and limited localization to intestinal target sites—factors that severely constrain its practical application.

To overcome these delivery challenges, the integration of synthetic biology and microbial engineering has emerged as a promising approach (18–22). Engineered probiotic platforms—especially *Escherichia coli* Nissle 1917 (EcN), known for its excellent safety profile and intestinal colonization capacity—have been widely employed for the oral expression and delivery of therapeutic peptides or proteins (23, 24). By incorporating functional peptide genes into EcN and regulating their expression via environment-responsive promoters, localized and inducible peptide expression can be achieved within the gut (25, 26). This strategy not only circumvents proteolytic degradation associated with oral delivery but also enhances the local concentration of the therapeutic molecule at the site of action, thereby potentiating its biological efficacy (23, 27, 28).

In this study, we established a targeted delivery system for the Scy peptide based on engineered EcN (EcN-Scy), utilizing the pNirB promoter, which is highly sensitive to anaerobic conditions, to drive site-specific expression in hypoxic regions of the intestine. We systematically evaluated the *in vitro* expression efficiency, genetic stability, and environmental resilience of EcN-Scy, followed by *in vivo* assessments in mice to determine its effects on growth performance, intestinal antioxidant capacity, inflammatory status, and microbiota homeostasis.

Furthermore, to explore its therapeutic potential in a pathogenic context, we employed a murine infection model challenged with enterotoxigenic *E. coli* K88 (*ETEC* K88). The ability of EcN-Scy to alleviate diarrhea, restore colonic mucosal integrity and modulate local immune responses was comprehensively investigated. Our findings provide strong support for the development of engineered microbial systems for targeted *in vivo* delivery of functional peptides, offering both theoretical insights and technical foundations for the creation of orally administered immunotherapeutic products for intestinal disorders.

## 2 Materials and methods

### 2.1 Strains, plasmids, and functional peptide genes

The expression host used in this study was EcN, preserved in our laboratory. The cloning host was chemically competent *E. coli* DH5α cells (Tsingke Biotechnology, Beijing). The expression plasmid was the anaerobically inducible pNirBMisl vector (abbreviated as pNir), which contains the pnirB promoter and a surface anchor protein gene Misl. The expression vector pNir was also obtained from our laboratory collection. The Scy and GFP genes were codon-optimized and synthesized *de novo* by Beijing Qingke Biotechnology Co., Ltd (**Table 1**).

**Table 1:** The optimized nucleotide sequences of Scy and GFP.

Gene	Sequence
Scy	GGCCAGGCACTGAATAAACTGATGCCTAAAATTGTGAGCGCAATTATCTATATGGTTGGTCAGCCGAATGCAGGTGTTACGTTTCTGGGTCATCAGTGTCTGGTTGAAAGTACCCGCCAGCCAGATGGTTTTTATACCGCAAAAATGTCATGTGCAAGTTGGACACATGATAATCCGATCGTTGGTGAAGGTCGTAGCCGTGTTGAACTGGAAGCCCTGAAAGGTAGCATTACCAATTTTGTTCAGACCGCAAGCAATTATAAAAAGTTTACAATCGATGAAGTGGAAGATTGGATTGCAAGCTAT
GFP	ATGCGCAAAGGTGAAGAACTGTTTACCGGTGTGGTTCCGATTCTGGTTGAACTGGATGGTGATGTTAATGGTCATAAATTTAGCGTTCGCGGTGAAGGTGAAGGTGATGCAACGAATGGTAAACTGACACTGAAATTTATTTGCACCACCGGTAAACTGCCTGTGCCGTGGCCTACCCTGGTGACGACACTGACCTATGGTGTTCAGTGTTTTGCGCGTTATCCGGATCATATGAAACAGCATGATTTTTTTAAAAGCGCCATGCCGGAAGGTTATGTTCAGGAACGTACCATTTCATTTAAAGATGATGGCACCTATAAAACCCGTGCCGAAGTTAAATTTGAAGGTGATACCCTGGTTAATCGTATTGAACTGAAAGGCATTGATTTTAAAGAAGATGGTAACATCCTGGGTCATAAACTGGAATATAATTTTAACAGCCATAACGTATATATCACGGCAGATAAACAGAAAAATGGCATTAAAGCAAACTTTAAAATCCGTCATAACGTGGAAGATGGCAGCGTCCAGCTGGCAGATCATTATCAGCAGAATACACCGATCGGTGATGGTCCAGTACTGCTGCCAGATAACCATTATCTGAGTACCCAGAGCGTTCTGAGCAAAGATCCGAATGAAAAACGTGATCATATGGTTCTGCTGGAATTTGTTACAGCCGCCGGTATTACACATGGTATGGATGAACTGTATAAA

### 2.2 Construction of recombinant plasmids and preparation of engineered strains

PCR Amplification and Vector Construction: Specific primers were designed to amplify the Scy, GFP, and GFP-Scy fusion gene fragments (**Table 1**). High-fidelity DNA polymerase (Vazyme Biotech) was used for PCR amplification. PCR products were verified by agarose gel electrophoresis and purified using a gel extraction kit. The pNir vector was linearized by digestion with XmaI and EcoRI (TaKaRa), and the target fragments were inserted via seamless homologous recombination, yielding recombinant plasmids pnir-Scy and pnir-GFP-Scy. Transformation and Screening of Recombinant Strains: The recombinant plasmids were first transformed into DH5α competent cells. Positive colonies were selected on ampicillin-containing agar plates and verified by colony PCR and sequencing. Verified plasmids were extracted and electroporated into EcN competent cells, resulting in the engineered strains EcN-Scy and EcN-GFP-Scy. The expression vector pNirBMisl (pNir) was also obtained from our laboratory collection.

### 2.3 Detection of recombinant protein expression

The engineered strains were inoculated into LB broth and cultured at 37°C with shaking at 200 rpm until reaching the logarithmic growth phase. Cells were then transferred to anaerobic incubation bags and cultured statically for 24 hours to induce expression via the pnirB promoter. Confocal Laser Scanning Microscopy: After induction, bacterial cells were washed three times with PBS and fixed with 4% paraformaldehyde. Fixed samples were mounted on slides and examined using a confocal laser scanning microscope to assess GFP expression. Quantification of Fluorescence Expression: A multimode microplate reader was used to measure GFP fluorescence intensity at different OD_600_ values. A linear regression curve was plotted to analyze the relationship between fluorescence intensity and bacterial cell density.

### 2.4 Stress tolerance assays of engineered strains

The growth characteristics and environmental tolerance of EcN, EcN-pnir, and EcN-Scy were evaluated under various stress conditions. Growth Curve Analysis: Each strain was inoculated into LB liquid medium, and the OD_600_ was measured at 2-hour intervals. Growth curves were plotted based on OD_600_ values over time. Heat Tolerance: Bacterial cultures were exposed to 65°C for 30, 40, 50, and 60 seconds, then rapidly cooled. Viable bacteria were quantified by plating on LB agar, and survival rates were calculated. Acid Tolerance: Each strain was inoculated into acidified LB media adjusted to pH 1.5, 2.0, and 2.5. Cultures were incubated at 37°C with shaking for 2 hours. OD_600_ values were recorded before and after treatment to calculate relative survival rates. Bile Salt Tolerance: Strains were cultured in LB medium supplemented with graded concentrations of bovine bile salts (0.5%, 1.0%, and 1.5%). After 12 hours of incubation, OD_600_ was measured to assess bile salt tolerance. Simulated Gastric and Intestinal Fluid Tolerance: Bacterial suspensions were first incubated in simulated gastric fluid (Shanghai Yuanye Bio-Technology Co., Ltd. Cat. No. R30388) for 2 hours, followed by transfer into simulated intestinal fluid (Shanghai Yuanye Bio-Technology Co., Ltd. Cat. No. R28753) for an additional 4 hours. Samples were taken at 0, 2, and 4 hours for colony-forming unit (CFU) counts to assess viability throughout the gastrointestinal simulation.

### 2.5 Animal experiment design and sample collection

Eight-week-old male BALB/c mice (n = 15) were purchased from Beijing Vital River Laboratory Animal Technology Co., Ltd. and randomly divided into three groups: control, EcN, and EcN-Scy (n = 5 per group). Mice were orally gavaged once every two days for a total of 13 days with PBS, EcN, or EcN-Scy suspensions (1 × 10^8^ CFU/mL). Body weight and clinical symptoms were monitored throughout the experiment. On day 14, mice were euthanized for sample collection. Blood serum, heart, liver, spleen, lungs, kidneys, colon tissue, and colonic contents were harvested for further analysis. No antibiotics were administered to mice at any stage of this experiment.

### 2.6 Serum biochemistry and histological analysis

Serum biochemical parameters, including alanine aminotransferase (ALT), aspartate aminotransferase (AST), total bilirubin (TBil), creatinine (Crea), urea, uric acid (UA), triglycerides (TG), cholesterol (Chol), and glucose (Glu), were measured by a a Hitachi 7600‐020 automatic clinical chemistry analyzer (Hitachi). Colon and organ tissues were fixed, dehydrated, paraffin-embedded, and stained with hematoxylin and eosin (H&E). Histological morphology was examined under a Leica DM3000 Microsystem (Leica, Germany).

### 2.7 Antioxidant and inflammatory cytokine assays

Colonic tissues were homogenized, and the supernatants were collected for analysis. Commercial assay kits were used to measure catalase (CAT), glutathione peroxidase (GSH-Px), total antioxidant capacity (T-AOC), total superoxide dismutase (T-SOD), and malondialdehyde (MDA) levels from Wuhan Servicebio Technology CO., LTD. Inflammatory cytokines including TNF-α, IL-4, IL-2, IL-10, and MCP-1 were quantified using enzyme-linked immunosorbent assay (ELISA) kits from Wuhan Servicebio Technology CO.,LTD.

### 2.8 16S rRNA gene sequencing and bioinformatics analysis

16S rRNA gene sequencing and bioinformatics analysis are based on our previous articles (1, 29). Genomic DNA was extracted from colonic contents, and the V3–V4 regions of the bacterial 16S rRNA gene were amplified by PCR and sequenced on the Illumina platform. Bioinformatic analysis was conducted using the services of the Meiji BioCloud platform. Alpha diversity metrics (Chao, Shannon, Simpson, Sobs) and beta diversity (Principal Coordinates Analysis, PCoA) were calculated. Microbial composition profiles were visualized, and differential taxa were identified using Linear Discriminant Analysis Effect Size (LEfSe) with a threshold LDA score > 3.5.

### 2.9 Construction of the ETEC K88 infection model

Mice were randomly divided into four groups: PBS control, K88 infection, K88 + EcN, and K88 + EcN-Scy (n = 8 per group). From day 0 to day 2, mice were orally gavaged once daily with either EcN (1 × 10^8^ CFU/mL, 200 μL), EcN-Scy (1 × 10^8^ CFU/mL, 200 μL), or PBS (200 μL). From day 3 to day 6, mice in the K88 infection groups received an additional daily gavage of ETEC K88 suspension (1 × 10^9^ CFU/mL, 200 μL). Diarrhea symptoms were monitored, and colonic tissues were collected for histopathological and inflammatory assessments. ETEC K88 is from our laboratory (30, 31). No antibiotics were administered to mice at any stage of this experiment.

### 2.10 Quantification of ETEC K88 eltA in colonic contents by qPCR

Frozen colonic luminal contents were collected at necropsy and stored at –80°C until analysis. For each mouse, approximately 100 mg of colonic content was used for bacterial DNA extraction using a commercial stool DNA extraction kit according to the manufacturer’s instructions. The concentration and purity of the extracted DNA were measured spectrophotometrically, and all samples were diluted with nuclease-free water to an equal DNA concentration across groups before qPCR. The eltA gene, encoding the A subunit of the heat-labile enterotoxin and specific for ETEC K88, was used as a marker of ETEC colonization in the colon. Quantitative PCR was performed on an Applied Biosystems 7500 Fast Real-Time PCR System (Foster City, CA, USA) using SYBR Green chemistry. Each 10 µL reaction contained 5 µL of 2× SYBR Green PCR Master Mix (Applied Biosystems), 0.5 µL of each forward and reverse eltA or faeG primer, 4 µL of normalized DNA template. The cycling protocol was: 50°C for 2 min, 95°C for 10 min, followed by 40 cycles of 95°C for 15 s and 57°C for 1 min. A melt-curve analysis was performed at the end of each run to confirm the specificity of the amplified product. No-template controls were included on each plate. Ct values for eltA or faeG were used to calculate the relative abundance of ETEC K88 in each sample. After normalization of DNA input across all samples, eltA or faeG levels in the ETEC K88, and ETEC K88+ECN-SCY groups were expressed as fold change relative to the PBS group, which was set to 1 and normalized to log_10_(1) = 0. The eltA primer was ATTAGCAGGTTTCCCACCGGATCA (forward) and TTGTGCTCAGATTCTGGGTCTCCT (reverse) (32); and the faeG primer was ACTCAGAAAACCTGATGGTGAAACT (forward) and CCCCACCTCTCCCTAACACA (reverse) (33).

### 2.11 Immunofluorescence and immunohistochemical staining

Paraffin-embedded tissue sections were deparaffinized, rehydrated, and subjected to antigen retrieval, followed by cooling to room temperature and rinsing with PBS. Non-specific binding sites were blocked using 5% bovine serum, and primary antibodies were applied: anti-MUC2 (Abcam, 1:200) and anti-ZO-1 (Invitrogen, 1:100), with overnight incubation at 4°C. After washing with PBS at room temperature the next day, corresponding fluorophore-conjugated secondary antibodies (Goat anti-Rabbit IgG, Alexa Fluor 488/594, 1:500) were added and incubated (34). Nuclei were counterstained with DAPI. Sections were mounted and imaged using a laser confocal microscope (Leica SP8).

### 2.12 Real-time qPCR

Total RNA was extracted with TRIzol, reverse-transcribed to cDNA, and amplified using SYBR Green (Roche) on a StepOnePlus real-time PCR system (35, 36). Gene-specific primers (**Table 2**; synthesized by Tsingke) were used. The relative mRNA expression of the target gene was analyzed by the 2−ΔΔCT method using the β‐actin as a normalization control.

**Table 2:** PCR primer sequence.

Gene	Primers (5’-3’) (F)	Primers (5’-3’) (R)
Scy	tatgtgcacacggacccgggGGCCAGGCACTGAATAAACTGA	tcgccatcttcatggaattcATAGCTTGCAATCCAATCTTCCA
GFP	tatgtgcacacggacccgggATGCGCAAAGGTGAAGAACTG	agtgcctggccTTTATACAGTTCATCCATACCATGTGTAA
GFP-Scy	tatgtgcacacggacccgggATGCGCAAAGGTGAAGAACTG	tcgccatcttcatggaattcATAGCTTGCAATCCAATCTTCCA
I1-1β	TGCCACCTTTTGACAGTGATG	ATGTGCTGCTGCGAGATTTG
Il-6	CCACTTCACAAGTCGGAGGCTTA	TGCAAGTGCATCATCGTTGTTC
Tnf-α	ACTCCAGGCGGTGCCTATGT	GTGAGGGTCTGGGCCATAGAA
Il-10	AAGGGTTACTTGGGTTGCCA	CCTGGGGCATCACTTCTACC
Zo-1	AACCCGAAACTGATGCTGTGGATAG	CGCCCTTGGAATGTATGTGGAGAG
Claudin	GCCATCTACGAGGGACTGTG	CCCCAGCAGGATGCCAATTA
β-actin	CGCAGCACTGTCGAGTC	TCATCCATGGCGAACTGGTG

### 2.13 Western blot analysis of GFP/GFP–Scy expression

EcN, EcN-GFP and EcN-GFP-Scy were grown to mid-log phase, and bacterial cells were collected by centrifugation (5,000 × g, 10 min, 4°C), washed once with ice-cold PBS and resuspended in lysis buffer. Protein concentration was determined using a BCA assay kit. Equal amounts of total protein were mixed with 5× SDS loading buffer, boiled, separated by SDS–PAGE and transferred onto PVDF membranes. After blocking with 5% skim milk in TBST, membranes were incubated overnight at 4°C with anti-GFP antibody, followed by secondary antibody. Bands were visualized using an ECL detection kit and a chemiluminescence imaging system.

### 2.14 Statistical analysis

All data were analyzed using one-way analysis of variance (ANOVA) with Duncan’s multiple range test for *post hoc* comparisons, conducted in SPSS version 26.0. Results are expressed as mean ± standard deviation (Mean ± SD). Differences were considered statistically significant at P < 0.05. Figures and graphs were generated using GraphPad Prism version 10.0.

## 3 Results

### 3.1 Construction and expression verification of the engineered strain EcN-Scy

**Figure 1:**
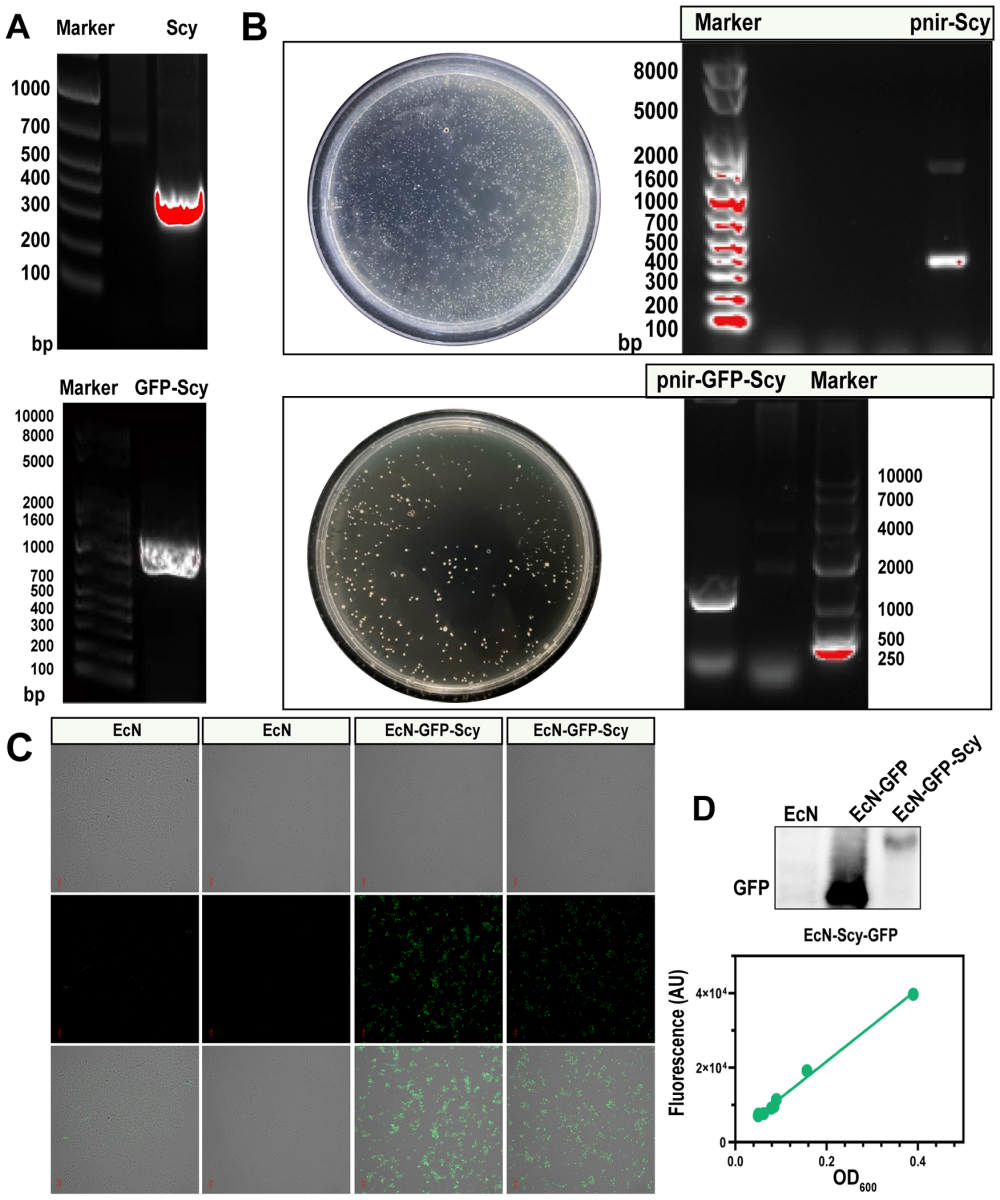
Construction and expression verification of engineered strain EcN-Scy. **(A)** PCR amplification of target genes Scy, and GFP-Scy; **(B)** Colony PCR identification of positive clones; **(C)** Confocal laser microscopy images of EcN and EcN-GFP-Scy; **(D)** Western bolt of GFP for EcN, EcN-GFP, and EcN-GFP-Scy; and Linear relationship between fluorescence intensity and OD_600_ of recombinant strain EcN-GFP-Scy.

The target gene Scy (306 bp), the green fluorescent protein gene GFP (714 bp), and the GFP-Scy fusion gene (306 + 714 = 1020 bp) were successfully amplified via PCR (**Figure 1A**). The recombinant plasmids pnir-Scy and pnir-GFP-Scy were confirmed as positive clones through ampicillin-resistant colony screening and colony PCR, and the correct insertion of sequences was validated by Sanger sequencing. The plasmids were subsequently electroporated into EcN, generating the engineered strains EcN-Scy and EcN-GFP-Scy (**Figure 1B**).

Confocal laser scanning microscopy revealed green fluorescence in EcN-GFP-Scy strains, while the control EcN strain exhibited no fluorescence signal (**Figure 1C**). Consistently, Western blot analysis detected a single GFP band at the expected molecular weight in EcN-GFP, whereas EcN-GFP-Scy showed a GFP-positive band of higher molecular weight, and no GFP signal was observed in wild-type EcN (**Figure 1D**). These results are fully in line with our plasmid design and provide protein-level confirmation that the GFP–Scy fusion is successfully expressed in EcN rather than merely cloned. Quantitative analysis demonstrated a significant linear correlation between fluorescence intensity and bacterial density (OD_600_) in EcN-GFP-Scy (**Figure 1D**), suggesting stable and efficient accumulation of exogenous proteins within the engineered strains.

### 3.2 Evaluation of biological properties and stress tolerance of EcN-Scy

**Figure 2:**
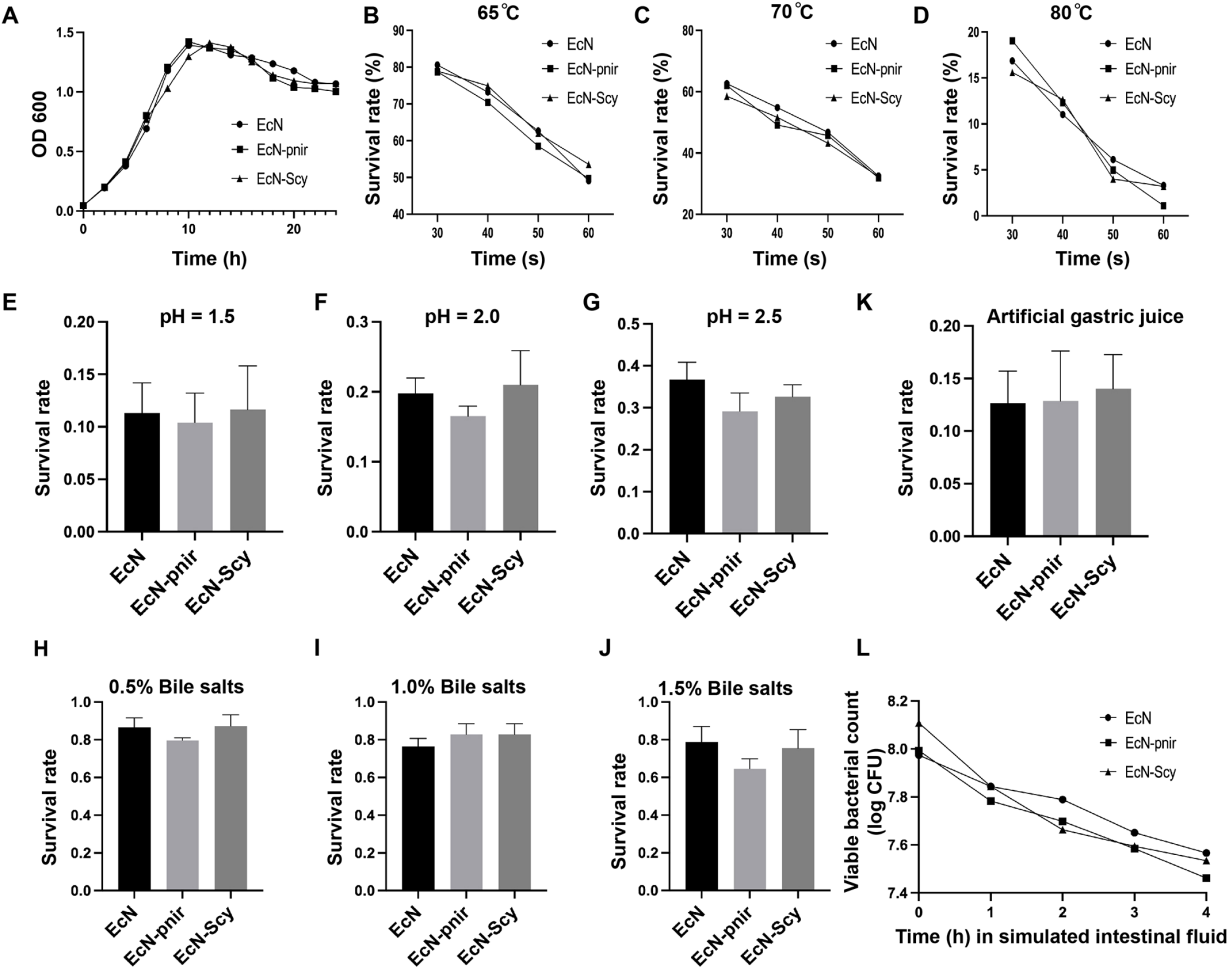
Biological characteristics and stress resistance of EcN-Scy strain. **(A)** 24 h growth curves of EcN, EcN-pNir, and EcN-Scy; **(B–D)** Survival rates of the three strains under different temperature and time treatments; **(E–G)** Survival rates under different pH conditions; **(H–J)** Survival rates under different bile salt concentrations; **(K, L)** Growth and survival of the three strains in simulated gastric fluid.

The growth curves of EcN-Scy, EcN-pnir, and wild-type EcN were highly similar (**Figure 2A**), indicating that expression of the Scy gene did not adversely affect bacterial growth. Upon exposure to 65°C for 30 seconds, all three strains maintained high survival rates, with maximum viability reaching 80.18% (**Figure 2B**). Further analysis showed a time- and temperature-dependent decrease in viability (**Figures 2B–D**), yet no significant differences were observed among the three strains, demonstrating that EcN-Scy exhibits excellent heat tolerance. Under acidic conditions (pH 2.5), survival rates for EcN, EcN-pnir, and EcN-Scy were 36.7%, 29.2%, and 32.7%, respectively (**Figures 2E–G**). Even under extreme acidity (pH 1.5), EcN-Scy maintained a survival rate of 28.2% (**Figure 2E**), comparable to that of the parental EcN strain, indicating strong acid resistance. In the presence of bile salts at concentrations of 0.5%, 1.0%, and 1.5%, all three strains maintained survival rates above 60% (**Figures 2H–J**), confirming the robust bile salt tolerance of EcN-Scy and its adaptability to simulated intestinal conditions. After 2 hours of treatment with simulated gastric fluid, no significant differences in survival rates were detected among the three strains (**Figure 2K**). Subsequent incubation in simulated intestinal fluid for 4 hours showed that EcN-Scy maintained a viable cell count of approximately 10^7^ CFU/mL (**Figure 2L**), indicating excellent gastrointestinal survivability and potential for oral administration.

### 3.3 Effects of EcN-Scy on mouse physiological functions

**Figure 3:**
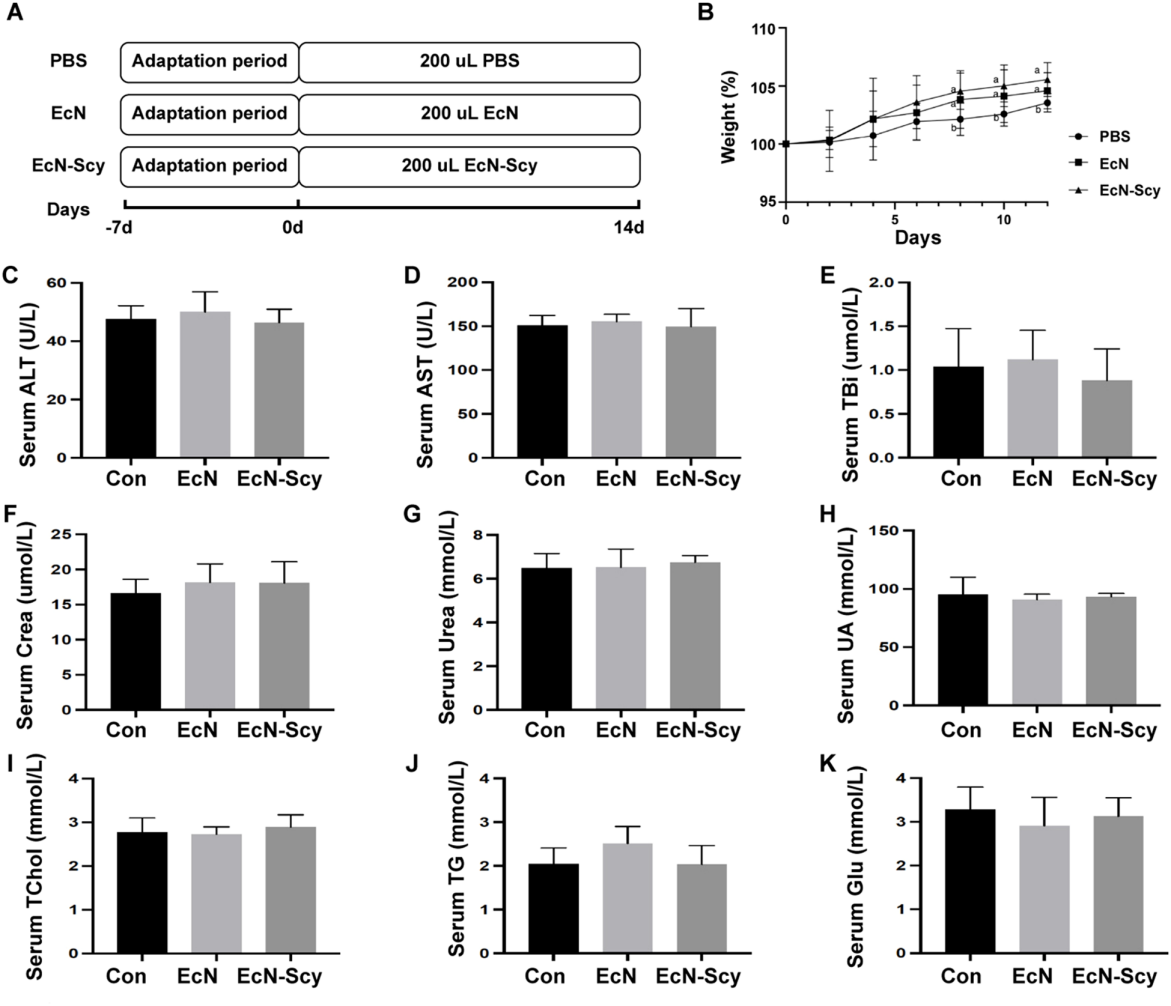
Effects of EcN-Scy on physiological functions in mice. **(A)** Schematic diagram of the experimental design; **(B)** Effect of EcN-Scy on body weight changes in mice; **(C–K)** Effects of EcN-Scy on serum physiological and biochemical parameters in mice.

Compared with the control group, both EcN and EcN-Scy treatments moderately promoted body weight gain in mice, although no significant difference was observed between the two treatment groups (**Figures 3A, B**). Regarding serum biochemical parameters, levels of alanine aminotransferase (ALT), aspartate aminotransferase (AST), total bilirubin (TBil), creatinine (Crea), urea, uric acid (UA), cholesterol (Chol), triglycerides (TG), and glucose (Glu) were not significantly altered by EcN-Scy administration (**Figures 3C-K**), suggesting that the engineered strain does not impair hepatic or renal function, nor does it disturb systemic metabolic homeostasis.

### 3.4 Protective effects of EcN-Scy on colonic function in mice

**Figure 4:**
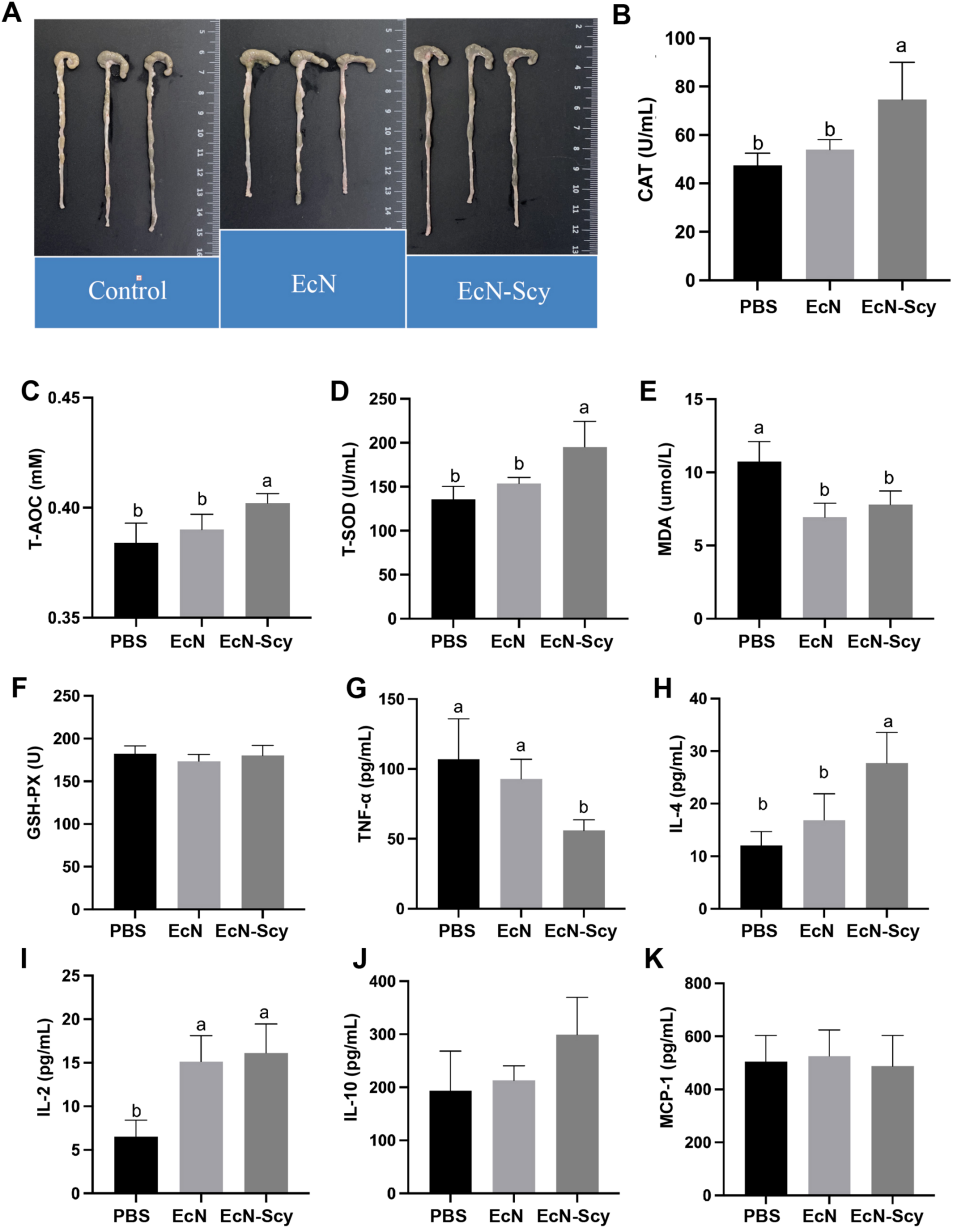
Improvement of colonic function in mice by EcN-Scy. **(A)** Effect of EcN-Scy on colonic length in mice; **(B–F)** Effects of EcN-Scy on antioxidant parameters in colonic tissues; **(G–K)** Effects of EcN-Scy on cytokine levels in colonic tissues.

Regarding colonic morphology, mice in the EcN-Scy group exhibited significantly longer colon lengths compared to the EcN group, while no significant difference was observed relative to the control group (**Figure 4A**). In terms of antioxidant capacity, the activities of catalase (CAT), total antioxidant capacity (T-AOC), and total superoxide dismutase (T-SOD) in colonic tissue were significantly elevated in the EcN-Scy group, while levels of malondialdehyde (MDA) were markedly reduced, indicating that EcN-Scy confers strong antioxidant protection in the colon (**Figures 4B-F**). Regarding inflammatory cytokine expression, EcN-Scy significantly downregulated the pro-inflammatory cytokine TNF-α and upregulated the anti-inflammatory cytokines IL-4 and IL-2 (**Figures 4G-I**). A trend toward increased IL-10 expression was also observed, while MCP-1 levels remained unchanged (**Figures 4J, K**). These findings suggest that the anti-inflammatory effect of EcN-Scy is primarily mediated through modulation of Th1/Th2 immune balance.

### 3.5 Modulatory effects of EcN-Scy on gut microbiota composition in mice

**Figure 5:**
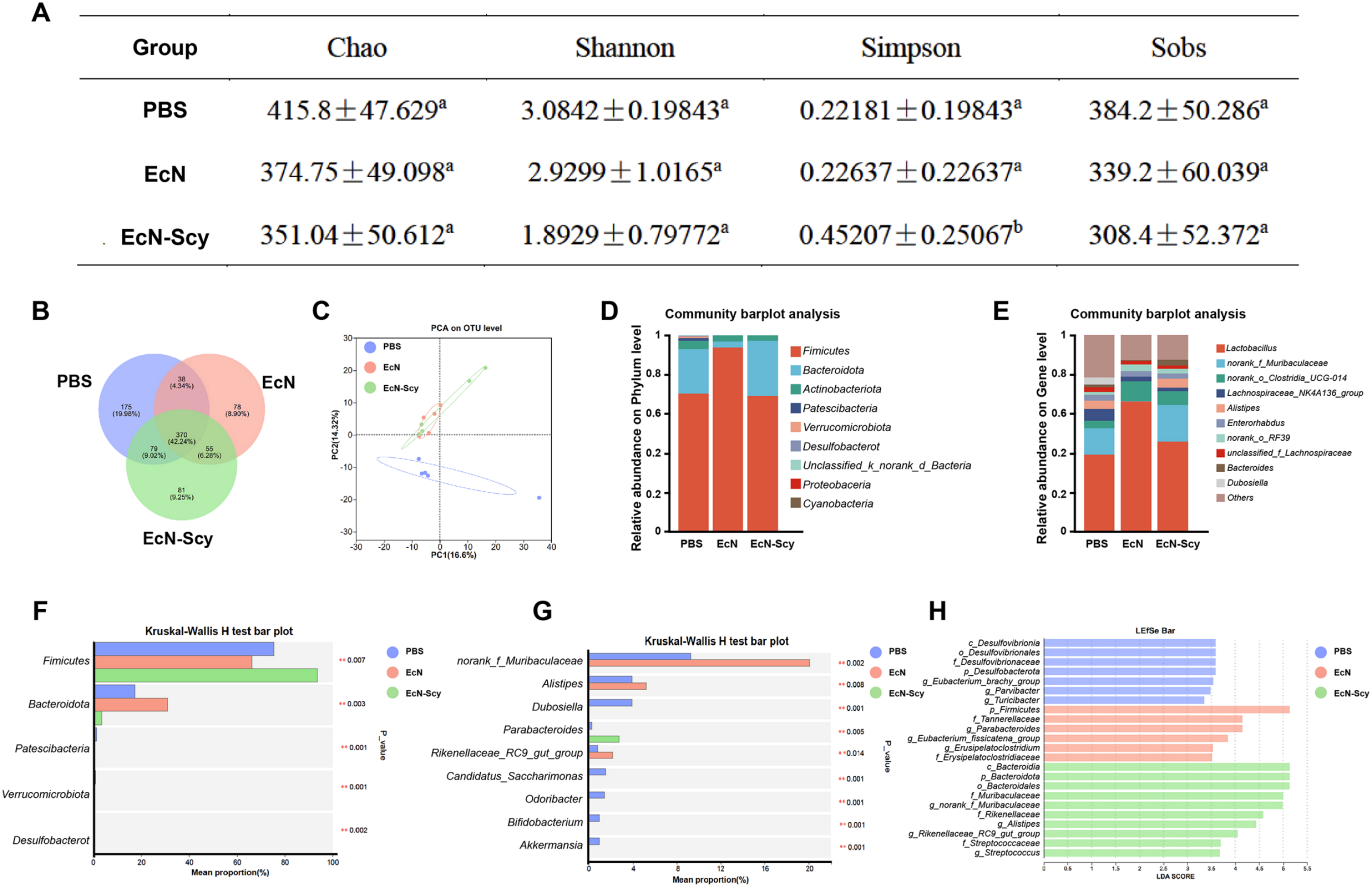
Modulatory effects of EcN-Scy on gut microbiota in mice. **(A)** Effect of EcN-Scy on α-diversity of colonic microbiota in mice; **(B)** Venn diagram of microbial community composition; **(C)** PCA analysis of microbial community structure; **(D)** Effect of EcN-Scy on colonic microbiota at the phylum level; **(E)** Effect of EcN-Scy on colonic microbiota at the genus level; **(F)** Differential taxa at the phylum level; **(G)** Differential taxa at the genus level; **(H)** LefSe analysis of differential microbial taxa at multiple taxonomic levels.

16S rDNA sequencing revealed that Chao, Shannon, and Sobs indices showed a decreasing trend following intervention with both EcN and EcN-Scy, indicating reduced microbial richness and evenness (**Figure 5A**). Notably, the Simpson index was significantly elevated in the EcN-Scy group (P < 0.05) (**Figure 5A**), suggesting a more concentrated microbial community structure. While partial overlap existed between the EcN and EcN-Scy groups, principal coordinates analysis (PCoA) further demonstrated a distinct separation in microbial composition between the EcN-Scy and control groups (**Figures 5B, C**). At the phylum level, EcN-Scy treatment significantly increased the relative abundance of *Firmicutes* (P < 0.01), while significantly reducing the abundance of potentially pathogenic or conditionally pathogenic phyla including *Patescibacteria*, *Verrucomicrobiota*, and *Desulfobacterota* (P < 0.01) (**Figures 5D, F**). At the genus level, EcN-Scy significantly decreased the relative abundances of *norank_f:Muribaculaceae*, *Alistipes*, *Dubosiella*, *Rikenellaceae_RC9_gut_group*, *Candidatus_Saccharimonas*, *Odoribacter*, *Bifidobacterium*, and *Akkermansia* (P < 0.05), while markedly increasing *Parabacteroides* (P < 0.01) (**Figures 5E, G**). Importantly, *Parabacteroides* is known for its ability to produce short-chain fatty acids, succinic acid and secondary bile acid, conferring anti-inflammatory properties and contributing to intestinal barrier integrity and immune modulation (37–39). Further analysis using LEfSe identified microbial taxa with LDA scores >3.5 as significant discriminators among groups. At the phylum level, *Desulfobacterota, Firmicutes*, and *Bacteroidota* were the representative dominant phyla in the control, EcN, and EcN-Scy groups, respectively (**Figure 5H**). At the family level, *Desulfovibrionaceae* (sulfate-reducing bacteria) was enriched in the control group, *Tannerellaceae* in the EcN group, and *Muribaculaceae* in the EcN-Scy group (**Figure 5H**). At the genus level, *norank_f:Muribaculaceae* was enriched in the EcN-Scy group, *Parabacteroides* in the EcN group, and *Eubacterium_brachy_group* in the control group (**Figure 5H**).

### 3.6 EcN-Scy alleviates ETEC K88–induced diarrhea and intestinal barrier injury

**Figure 6:**
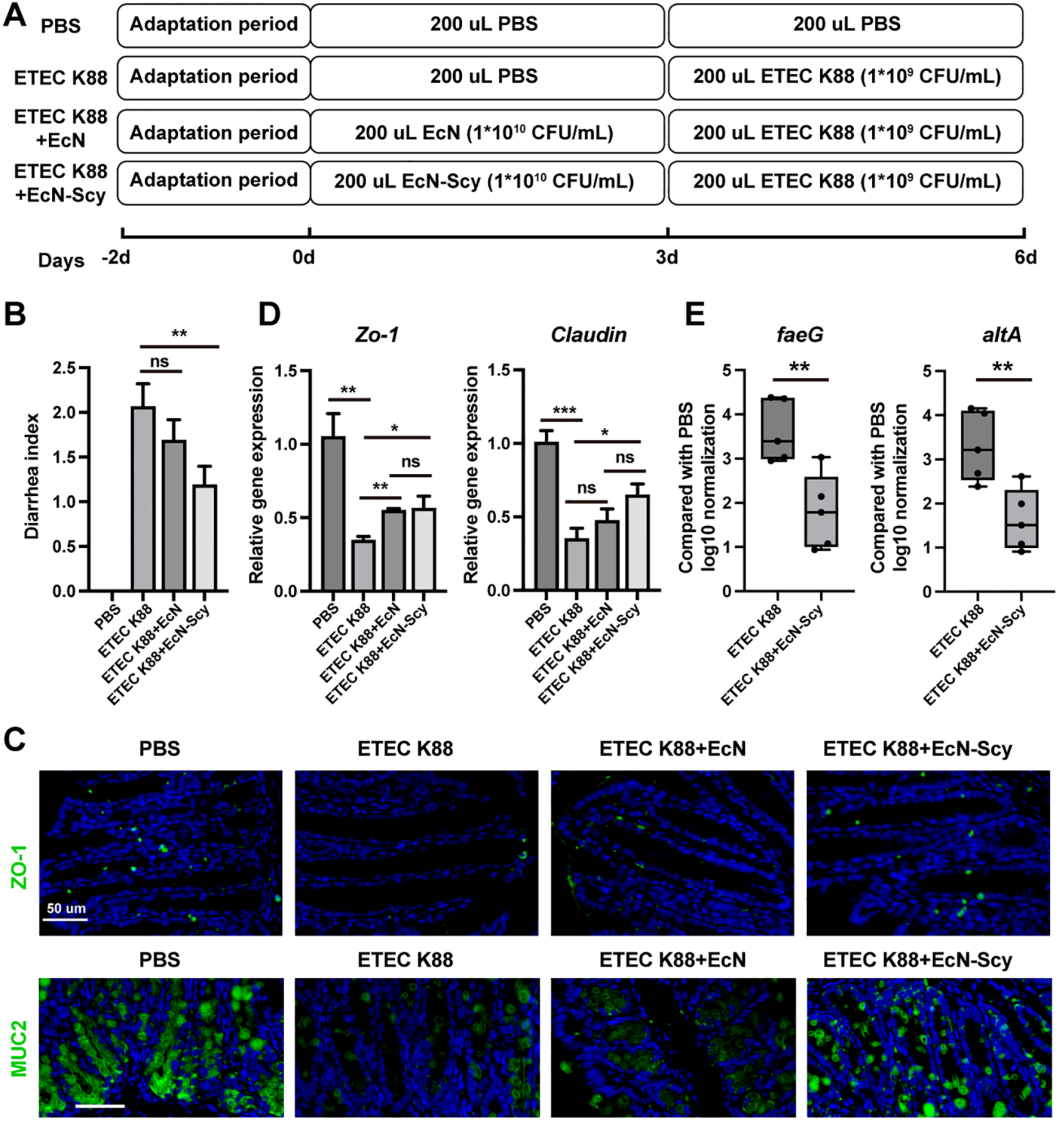
EcN-Scy alleviates diarrhea symptoms and intestinal barrier disruption induced by ETEC K88. **(A)** Schematic diagram of the experimental design; **(B)** Number of diarrheal mice; **(C)** Immunofluorescence analysis of ZO-1 and MUC2 expression in colonic tissues; **(D)** RT-qPCR analysis of ZO-1 and Claudin gene expression in colonic tissues; **(E)** Quantitative analysis of faeG and eltA gene abundance in colonic contents. *P < 0.05, **P < 0.01, ***P < 0.001, ns > 0.05.

To further assess the therapeutic potential of EcN-Scy against enteric pathogenic infection, we established a murine model of ETEC K88 challenge and assigned mice to four groups: PBS control, K88 infection, K88+EcN, and K88+EcN-Scy (**Figure 6A**). By administering repeated oral gavage for both modeling and intervention, we systematically evaluated the effects of each treatment on diarrheal symptoms and intestinal tissue architecture. Mice in the K88 group developed pronounced diarrheal phenotypes, including loose and watery stools and lethargy, with significantly elevated diarrhea scores (**Figure 6B**). EcN treatment partially ameliorated these symptoms, although they remained evident (**Figure 6B**). In contrast, EcN-Scy markedly reduced both the incidence and scores of diarrhea, with clearly improved clinical manifestations, indicating a stronger efficacy of EcN-Scy in mitigating ETEC K88–induced diarrhea (**Figure 6B**). Furthermore, immunofluorescence staining demonstrated that EcN-Scy significantly upregulated the expression of key epithelial barrier proteins ZO-1 and MUC2 (**Figure 6C**). Consistently, RT-qPCR analysis confirmed that EcN-Scy significantly increased the transcription of tight junction–related genes, including ZO-1 and Claudin (**Figure 6D**). The faeG and eltA genes encode the K88 (F4) fimbrial adhesin and the heat-labile enterotoxin, respectively, and are present in ETEC K88 but absent in EcN/EcN-Scy, making them highly specific marker genes that reflect the adhesion/colonization level of K88 (32, 33, 40, 41). By performing quantitative real-time PCR analysis of faeG and eltA in colonic contents, we found that the levels of both genes were markedly increased in the ETEC K88 group, whereas their abundance was significantly reduced in the K88 + EcN-Scy group (P < 0.01) (**Figure 6E**). These findings indicate that EcN-Scy not only colonizes the intestine but also exhibits a strong antagonistic effect against ETEC K88. Such antagonism may be associated with multiple mechanisms, including the antimicrobial activity of the surface-displayed Scy peptide on EcN-Scy, competition for colonization sites, modulation of the intestinal microenvironment, and activation of local immune responses, suggesting that EcN-Scy has considerable anti-pathogenic potential. Together, these data indicate that EcN-Scy confers robust protection on intestinal structural integrity and epithelial barrier function.

### 3.7 EcN-Scy attenuates ETEC K88–induced colonic inflammatory responses

**Figure 7:**
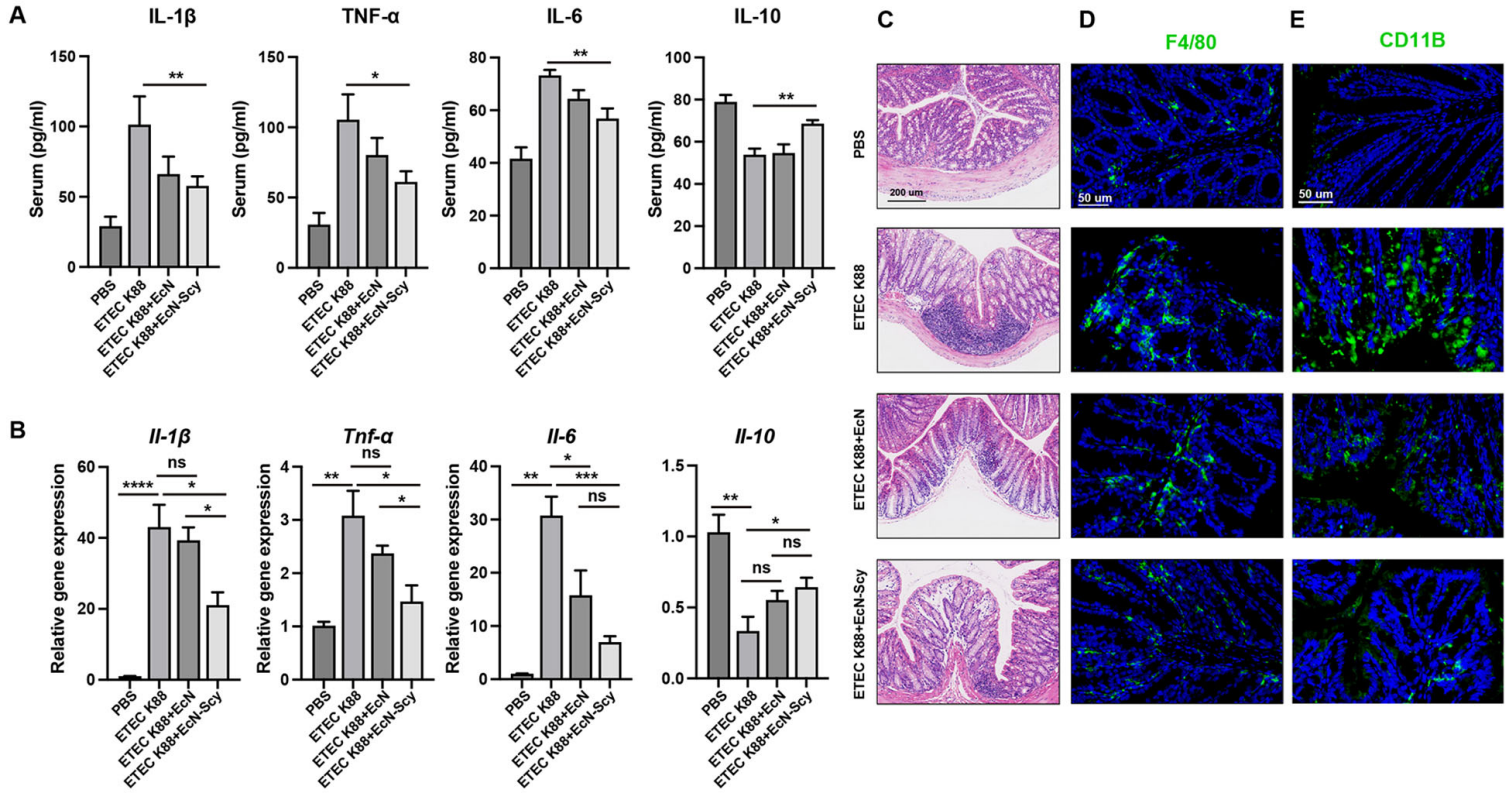
EcN-Scy alleviates colonic inflammatory responses induced by ETEC K88. **(A)** ELISA analysis of serum levels of IL-1β, TNF-α, IL-6, and IL-10; **(B)** RT-qPCR analysis of IL-1β, TNF-α, IL-6, and IL-10 gene expression in colonic tissues; **(C)** Histological assessment of colonic tissues by HE staining; **(D, E)** Immunofluorescence analysis of inflammatory cell infiltration in colonic tissues (F4/80 indicates macrophages, CD11B indicates myeloid immune cells). *P < 0.05, **P < 0.01, ***P < 0.001, ****P < 0.0001, ns > 0.05.

To investigate the anti-inflammatory effects of EcN-Scy, we quantified canonical inflammatory cytokines in colonic tissue and serum. ELISA results showed that K88 infection significantly increased the levels of pro-inflammatory cytokines IL-1β, TNF-α and IL-6, while markedly reducing the anti-inflammatory cytokine IL-10 (**Figure 7A**), indicative of a strong inflammatory response. In contrast, EcN-Scy treatment significantly suppressed IL-1β, TNF-α and IL-6 while elevating IL-10, shifting the overall cytokine profile toward immune homeostasis (**Figure 7A**). RT-qPCR analyses further validated these findings at the transcriptional level: IL-1β, TNF-α and IL-6 mRNA expression in colonic tissue was significantly downregulated in the EcN-Scy group (**Figure 7B**), consistent with the ELISA data. In addition, H&E and immunofluorescence staining revealed massive inflammatory cell infiltration in the K88 group, which was markedly reduced following EcN-Scy intervention, underscoring its potent anti-inflammatory activity (**Figures 7C-E**).

## 4 Discussion

In this study, we successfully constructed a genetically engineered strain of EcN-Scy that stably expresses the immunomodulatory peptide Scy, and systematically evaluated its *in vitro* stability, biosafety, and effects on intestinal health in a murine model. Our findings collectively demonstrate the promising potential of EcN-Scy for oral intestinal immunotherapy.

Firstly, in the construction of the engineered strain, the Scy gene was codon-optimized and inserted into an expression vector under the control of the anaerobically inducible promoter pNirB, enabling hypoxia-responsive expression of a GFP–Scy fusion protein in EcN. Previous studies have shown that the pNirB promoter is effectively activated in low-oxygen environments and confers strong intestinal targeting capacity (42–46). In line with these reports, our plasmid construction, PCR and sequencing analyses confirmed successful integration of the GFP and GFP–Scy fragments, and confocal microscopy revealed inducible and appropriately localized GFP–Scy fluorescence signals in EcN under anaerobic conditions, with fluorescence intensity showing a linear correlation with OD_600_, similar to the findings of Samadi et al. using GFP in the pNirB system (46). Together with the *in vivo* functional readouts—namely, attenuation of inflammation, promotion of barrier repair and protection against ETEC infection—these data provide converging, albeit indirect, evidence that EcN-Scy can efficiently express the Scy-containing fusion protein in the intestinal environment. We acknowledge that more direct, Scy-specific protein-level analyses (e.g., using a Scy-specific antibody or mass spectrometry) were not performed in the present study and represent an important technical limitation that will be addressed in future work. Secondly, EcN-Scy exhibited growth kinetics comparable to the parental EcN strain and maintained high survival rates under multiple stress conditions, including heat, acid, bile salts and simulated gastrointestinal fluids. This indicates that expression of the GFP–Scy fusion does not adversely affect the basic physiological properties of the host bacterium and supports its ability to survive and transiently colonize the gastrointestinal tract. Compared with conventional encapsulation or chemical modification strategies for peptide delivery, such an engineered expression system allows continuous, *in situ* production of functional molecules at the mucosal surface, thereby offering clear advantages in terms of delivery efficiency and site specificity (47–49).

In animal studies, EcN-Scy administration did not cause hepatic or renal toxicity or metabolic abnormalities, confirming its biosafety and compatibility. Furthermore, EcN-Scy significantly enhanced colonic antioxidant capacity, as evidenced by increased T-SOD, CAT, and T-AOC levels, and decreased MDA accumulation, suggesting its potential in relieving oxidative stress and maintaining intestinal homeostasis. These findings are consistent with previous reports of Scy’s intrinsic antioxidant activity in mariculture large yellow croaker *Larimichthys crocea (*50, 51). Additionally, EcN-Scy reduced pro-inflammatory TNF-α expression and elevated IL-4 and IL-10 levels, indicating its ability to modulate the colonic immune microenvironment and attenuate inflammatory responses. Prior studies have demonstrated that probiotic strains expressing small immunoregulatory peptides can enhance intestinal barrier function and alleviate inflammation (2, 52, 53); our work further extends this concept to the functional delivery of novel peptides via engineered probiotics.

Gut microbiota analysis revealed that EcN-Scy also exerts significant modulatory effects on intestinal microbial ecology. Treatment with EcN-Scy reduced microbial diversity (as reflected by Shannon and Chao indices) while increasing the Simpson index, indicating a shift toward a more focused community of beneficial dominant taxa. Importantly, no antibiotics were administered to mice at any stage of the experiment, and antibiotics were used only during *in vitro* strain construction and selection. Therefore, these microbiota changes are unlikely to be driven by antibiotic selection pressure and are more plausibly attributable to the activity of the engineered EcN-Scy strain itself. LEfSe analysis showed significant enrichment of *Parabacteroides*, alongside suppression of potentially pathogenic genera such as *Muribaculaceae* and *Alistipes*. Such compositional restructuring promotes an anti-inflammatory intestinal environment and supports barrier function recovery. These findings are consistent with other studies using engineered probiotics to reshape host microbiota—for example, Chen et al. demonstrated that EcN expressing anti-IL-6 nanobodies can modulate gut microbial communities and relieve colitis (53).

Mechanistically, our findings support a model in which EcN-Scy protects the intestinal barrier by intervening at multiple levels along the “pathogen burden–inflammation–tight junction” axis. At the upstream level, qPCR analysis of ETEC K88–specific virulence genes showed that the relative abundances of *faeG* and *eltA* were markedly increased in the K88 group but significantly reduced in the K88 + EcN-Scy group, indicating that EcN-Scy effectively lowers K88 adhesion and toxin load in the colon. Reduced exposure to K88 fimbrial and toxin signals is expected to attenuate activation of TLR/NF-κB–driven inflammatory pathways, thereby limiting the overproduction of TNF-α, IL-6 and IL-1β and restoring a more balanced cytokine profile characterized by increased IL-10 (54–56). Since pro-inflammatory cytokines and oxidative stress are known to disrupt the cytoskeleton and tight junction complexes, leading to downregulation and mislocalization of ZO-1, Occludin and Claudins (57, 58), the observed shift toward a less inflammatory mucosal environment likely contributes directly to the recovery of tight junction protein expression and localization. Thus, the concomitant reductions in faeG/eltA load, normalization of cytokine profiles and restoration of tight junction markers together suggest that EcN-Scy breaks the vicious cycle of “pathogen overgrowth–inflammatory amplification–barrier breakdown” and promotes the re-establishment of epithelial barrier integrity.

Taken together, our data support a model in which EcN-Scy alleviates ETEC K88–induced pathology through a combination of reduced pathogen burden, dampened mucosal inflammation, enhanced antioxidant defenses, and restoration of tight junction integrity. These mechanistic inferences are currently derived from selected readouts (cytokines, barrier proteins and microbiota composition) rather than unbiased, systems-level analyses, and we did not directly quantify Scy protein levels using Scy-specific antibodies or mass spectrometry. In future work, we plan to perform transcriptomic (RNA-seq) and proteomic profiling of colonic tissues from control, K88 and EcN-Scy–treated animals—together with more refined, protein-focused assays for Scy itself—to map global changes in signaling pathways, immune networks and epithelial barrier programs, and to identify key molecular targets underlying the protective effects of EcN-Scy.

In summary, within the context of this exploratory, proof-of-concept study, the engineered strain EcN-Scy exhibits excellent stability, stress resistance, safety, immunomodulatory activity and pathogen antagonism, highlighting its potential as a novel orally administered microbial therapeutic. We acknowledge that the sample size was relatively small, which may limit the ability to detect more subtle effects; nonetheless, EcN-Scy consistently improved diarrhea scores, histopathological damage, inflammatory cytokine profiles and barrier-related markers, supporting the robustness of the main conclusions. Future studies should further dissect the mechanisms of action of EcN-Scy, including its impact on intestinal epithelial stem cells, TLR-mediated signaling and bacterial adhesion/colonization, and incorporate dedicated comparisons between exogenous Scy administration and EcN-Scy–mediated delivery to distinguish the contributions of the peptide versus the probiotic platform. Expanding sample size and employing additional animal models, particularly target livestock species such as pigs, will be essential to enhance statistical power, verify generalizability and provide stronger evidence for translational and industrial applications.

## Data Availability

The datasets presented in this study can be found in online repositories. The names of the repository/repositories and accession number(s) can be found below: https://www.ncbi.nlm.nih.gov/, accession number PRJNA1312035.
